# An Innovative Medical Image Analyzer Incorporating Fuzzy Approaches to Support Medical Decision-Making

**DOI:** 10.3390/medsci13030097

**Published:** 2025-07-24

**Authors:** Cristina Ticala, Camelia M. Pintea, Mihaela Chira, Oliviu Matei

**Affiliations:** Department of Mathematics and Computer Science, North University Center at Baia Mare, Technical University Cluj-Napoca, 400114 Cluj-Napoca, Romania

**Keywords:** image processing, magnetic resonance imaging, MRI, fuzzy logic, artificial intelligence, medical platform, biomedical imaging, brain imaging

## Abstract

Background/Objectives: This paper presents a medical image analysis application designed to facilitate advanced edge detection and fuzzy processing techniques within an intuitive, modular graphical user interface. Methods: Key functionalities include classical edge detection, Ant Colony Optimization (ACO)-based edge extraction, and fuzzy edge generation, which offer improved boundary representation in images where uncertainty and soft transitions are prevalent. Results: One of the main novelties in contrast to the initial innovative Medical Image Analyzer, iMIA, is the fact that the system includes fuzzy C-means clustering to support tissue classification and unsupervised segmentation based on pixel intensity distribution. The application also features an interactive zooming and panning module with the option to overlay edge detection results. As another novelty, fuzzy performance metrics were added, including fuzzy false negatives, fuzzy false positives, fuzzy true positives, and the fuzzy index, offering a more comprehensive and uncertainty-aware evaluation of edge detection accuracy. Conclusions: The application executable file is provided at no cost for the purposes of evaluation and testing.

## 1. Introduction

Nowadays, medical image analyzers are advanced computational tools designed to assist doctors in order to take argumentative decisions well regarding medical diagnostics in the personalized case of each patient [1,2]. The new technologies such as deep learning in general artificial intelligence methods [3,4,5,6] enhance the accuracy and consistency of image interpretation. Overall, the medical staff, including radiologists, could better understand magnetic resonance imaging (MRI), computer tomography (CT), X-ray, and ultrasound results.

Medical image analyzers are used to interpret, quantify, and enhance medical images [7,8]. These systems typically integrate a range of image processing techniques to facilitate the detection, segmentation, and analysis of anatomical structures and pathological regions. The primary objective of medical image analysis software is to improve diagnostic accuracy, support clinical decision-making, and enhance the reproducibility of image interpretation.

Mehta et al. [9] provide an AI-powered medical image analyzer, called *MedSeg* [10], in order to efficiently and accurately identify chest diseases based on chest X-rays. The authors use multiple advanced image processing methods and machine learning algorithms by integrating the Inception V3 model (Google deep convolutional neural network DCNN [11,12]) into training a deep neural network to recognize as many patterns of different medical afflictions as possible.

Recently, in 2025, the *RenalSegNet* deep learning-based framework was proposed [13] to enhance automated segmentation of renal tumors, veins, and arteries in contrast-enhanced CT scans. The application has good accuracy and therefore a positive effect on renal cancer treatment and on preoperative planning and postoperative evaluation.

Large Language Models (LLMs) and Multimodal Language Models (MLLMs) [14,15] are some of the newest methods used in image analysis. MedSeg-R [14], Reasoning Segmentation in Medical Images, incorporates both LLM and MLLM interpreters for clinical questions and an efficient mechanism to produce segmentation masks for medical images.

Classical operators continue to be employed while developing new techniques for medical image segmentation. For example, Ramya et al.’s  [16] proposal uses Log, Zero-Cross, Sobel, and Canny [17] operators to detect brain tumor edges.

A recent study [18] explores how particles move when their ability to spread out changes exponentially over time depending on whether they speed up or slow down. This is in the context of a diffusion process with a time-dependent diffusion coefficient exponentially increasing and decreasing in time. The study also specified that the findings could be used in medicine, for example, on how water diffuses in brain tissues, which can exhibit all three types of time-dependent diffusion behaviors studied. Based on these considerations, Liao et al. [19] show that diffusion MRI can reveal microstructural changes in brain tissue as it develops or gets affected by disease. They also demonstrate that their machine learning model can quickly and accurately pick up these subtle cellular details. In recent years, there has been a growing interest in biophysical models that directly parameterize tissue microgeometry, enabling the quantification of changes in health and disease (e.g., MRI diffusion, Alzheimer’s disease). Specifically, for white matter (WM), the Standard Model (SM) has been proposed as a comprehensive framework by Novikov et al. [20,21,22].

Ghosh et al. [23] address Alzheimer’s disease by developing a fuzzy-based MRI segmentation method. Their method improves contrast in key brain regions, accurately detects edges, and enhances boundary detection. Testing on ADNI images [24] showed promising results in better identifying brain structures related to the disease.

Fuzzy logic is a mathematical framework that models uncertainty and partial truth, enabling systems to handle imprecise, vague, or incomplete information more effectively than classical binary logic [25,26,27]. Unlike crisp systems that rely on strict true or false decisions, fuzzy logic allows degrees of membership, offering a more flexible and human-like reasoning approach. In medical image analyzers, fuzzy techniques are particularly valuable for processing images with low contrast, noisy backgrounds, or ambiguous boundaries, as they can better represent the gradual transitions between tissues. By incorporating fuzzy edge detection [28,29,30] and fuzzy region classification [31,32,33], medical analyzers can improve the robustness and sensitivity of diagnostic tools, especially in complex imaging modalities where traditional methods may fail to capture subtle pathological changes.

A comparison of classical operators (Sobel, Prewitt, Roberts, and Canny) versus fuzzy edge detection methods (Type-1 and Type-2 fuzzy logic) is presented in [34]. In order to handle uncertainty and imprecision in medical data, fuzzy logic is applied to medical analyzers. For example Shoo et al. [35] proposed a MultiTumor Analyzer (MTA-20–55), a network for efficient classification of detected brain tumors from MRI images, incorporating the Bayesian fuzzy clustering technique for brain image segmentation.

Farnoosh and Noushkaran [36] developed an unsupervised pseudo-deep approach for brain tumor detection in magnetic resonance images. The unsupervised pseudo-deep method identifies the tumor area through spectral Co-Clustering, and this area is used as an input for the fuzzy C-means [37] to extract a refined tumor image.

Ant Colony Optimization (ACO) [38] is a clever bio-inspired algorithm based on how real ants find their way around using pheromone trails. ACO mimics this process to solve complex optimization problems. The results of applying ACO to medical diagnosis [39,40] and radiological imaging [41,42,43] (e.g., image segmentation, edge detection) are promising; it shows how metaheuristics, including bio-inspired algorithms, can help in accurate and efficient decision-making in healthcare [44,45].

The authors’ first version of the *innovative Medical Image Analyzer*, *iMIA,* application [46] was successfully tested on COVID-19 lung images. The filtering phase could use Roberts, Prewitt, Sobel, and Laplacian mask operators [47,48]. iMIA uses diverse algorithms, both traditional and AI-based methods, for image edge detection [49,50,51]. The current extended new and interactive GUI version includes the fuzzy approach.

The current version of the application introduces a restructured interface compared to the initial *iMIA* implementation [46]. While the earlier version was organized into two main tabs, namely “Edge Detection” and “Filter & Analysis,” the updated version adopts a modular, window-based architecture. From a centralized main window, users can access dedicated processing modules by selecting the corresponding function buttons. The primary entry point of the application is the “Load Image” button, which allows the user to import a medical image that serves as the basis for all subsequent analyses. The core processing capabilities from the initial version have been retained—excluding the pixel count chart. Several advanced features have been added to enhance image interpretation and user interaction and are the main paper-work objectives.

O1 The use of the Fuzzy Logic Matlab toolbox, R2023a enhancing iMIA features during image analysis.
O2 The use of fuzzy indexes computed with Canny edge [52] image results as ground truth when compared with other methods. ACO with fuzzy metrics was considered here based on comparative metrics (e.g., Jaccard index, Figure of Merit) results from [53]. More comprehensive statistical visualization replaced the pixel count chart. Four separate charts have been introduced to display fuzzy metrics, which present fuzzy false negatives (FFN), fuzzy false positives (FFP), fuzzy true positives (FTP), and the fuzzy index (validated metrics [53,54]).
O3 Fuzzy processing: The application now supports the generation of both fuzzy edge and linked fuzzy edge of the image. This module offers the possibility to save fuzzy edges.
O4 Clustering: Fuzzy C-means image clustering has been incorporated to facilitate soft tissue classification.
O5 The zoom image and edges module offers interactive zooming and panning capabilities, allowing detailed inspection of the processed image with the option to overlay Canny edges for comparative visualization.
O6 Comparison of ACO with U-Net [55] in extracting brain boundaries from medical image such as MRI or CT scans.

Regarding the article organization, after the preliminaries, the main part of the article is detailed in Section 2; here is presented the extended *innovative Medical Image Analyzer* (*iMIA*) platform with the fuzzy approach. Section 3, Numerical Examples, starts with the medical dataset presentation and the hardware and software utilities used to obtain the presented results. Section 4 follows with discussion and image analysis on specific fuzzy results and an additional U-Net vs. ACO comparison. Section 5 summarizes the fuzzy influence over *iMIA* platform results and several future possible improvements.

## 2. Materials and Methods

The versatility of MATLAB [56,57,58] makes it well suited for the rapid prototyping and deployment of interactive applications that integrate complex algorithms with user-friendly visualization tools. In the context of medical imaging, MATLAB provides robust support for advanced processing techniques, such as edge detection, fuzzy logic, and clustering, which are essential for analyzing images with rather high noise, low contrast, and ambiguous anatomical boundaries [59,60,61].

The application developed leverages MATLAB’s capabilities to offer a modular, interactive system which allows users to efficiently load, process, and analyze medical images using both classical and fuzzy-based techniques.

### 2.1. Proposal: Fuzzy Approach of the Innovative Medical Image Analyzer

The developed medical image analysis application builds upon MATLAB’s powerful computational framework to deliver an interactive, modular tool specifically designed for advanced edge detection and fuzzy image processing. The application enables users to load a medical image and explore a variety of processing methods within dedicated windows accessible through the main interface.

In addition to classical edge detection and Ant Colony Optimization (ACO)-based methods [38], the application introduces fuzzy edge detection and fuzzy edge linking to address the common challenge of soft and uncertain boundaries in medical images. Furthermore, it incorporates fuzzy C-means clustering [37] to facilitate unsupervised tissue classification, offering a flexible alternative to hard segmentation techniques. A dedicated Zoom and edge visualization module provides detailed image inspection with interactive zooming, panning, and edge overlay capabilities. The system also includes a comprehensive fuzzy performance analysis, where four key metrics—fuzzy true positives, fuzzy false positives, fuzzy false negatives, and the fuzzy index—are graphically represented to offer a more nuanced evaluation of the image processing outcomes. Overall, this application presents a versatile and intuitive platform that enhances the interpretability and precision of medical image analysis through the integration of classical and fuzzy-based methods.

#### 2.1.1. iMIAStructure and Functional Organization

**Figure 1:**
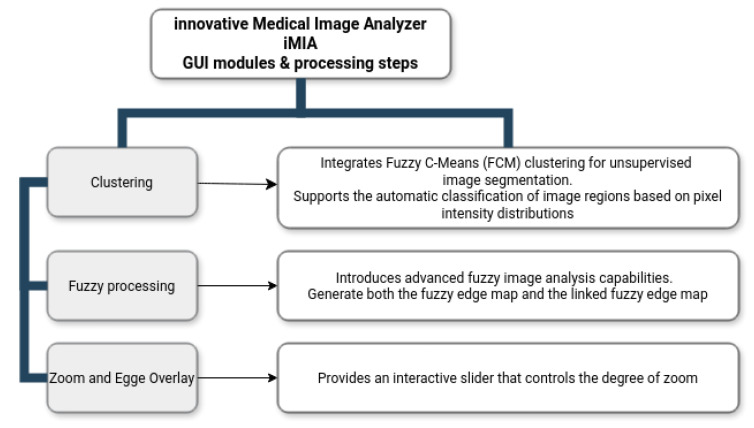
*Innovative Medical Image Analyzer*, *iMIA*, workflow.

The new and improved version of the innovative Medical Image Analyzer application is designed with a modular and user-friendly structure, where the primary operations are accessible from a central control window. The main interface provides a clear and logical workflow that enables users to sequentially load images, process them, and analyze results within dedicated functional windows. The *iMIA* workflow is presented in Figure 1.

#### 2.1.2. Edge Generation and Performance Analysis

A key component of the application is the Generate Edges functionality, which enables the user to extract edges from the loaded image and visualize them within the same window as the processed image. The edge detection method can be selected from a drop-down menu offering both classical and advanced techniques, including Prewitt, Sobel, Roberts, Canny, as well as four ACO-based methods, ACO_KH, ACO_Chi, ACO_sin, and ACO_pow using four different operators as in [49,50]. The interface allows the simultaneous generation of up to four edges, providing the capability to systematically compare classical edge detection algorithms with ACO-based approaches.

Beneath the edge visualizations, the application presents four fuzzy performance charts: fuzzy false negatives (FFN), fuzzy false positives (FFP), fuzzy true positives (FTP), and the fuzzy index (FI) (validated metrics [53,54]). These charts quantify the performance of the selected edge detection methods, using the Canny edge as the reference ground truth for comparison. This structure enables a direct visual and statistical evaluation of both the quality and the uncertainty of the detected edges.

#### 2.1.3. Filtering and Edge Detection on Filtered Images

The Filters module provides an interactive environment for analyzing the effects of image enhancement techniques on edge detection. When the Filters button is activated, the performance charts are temporarily hidden, and the interface displays four image placeholders. Two of these placeholders present the original loaded image, each of which can be independently processed using different filters selected from a drop-down menu. Available filters include Laplace, Sobel Gradient, Smoothed Sobel Gradient, and Sharpen. The corresponding edges for the filtered images are displayed in the adjacent placeholders, allowing users to assess the effect of filtering on edge extraction. The edge detection method for both filtered images is selected from a shared drop-down list containing the same eight techniques as in the edge generation module.

#### 2.1.4. Interactive Zoom and Edge Overlay

The Zoom Image and Edges module offers detailed, dynamic inspection of the processed images. This window provides an interactive slider that controls the degree of zoom, enabling both magnification and real-time panning of the image. Users can choose to overlay the extracted edges onto the original image and adjust the zoom level to focus on specific regions of interest. The design ensures that users can navigate the image smoothly and analyze the precision of edge localization at various scales.

### 2.2. Clustering

The Clustering module integrates fuzzy C-means (FCM) clustering for unsupervised image segmentation [37]. This functionality supports the automatic classification of image regions based on pixel intensity distributions while accommodating partial membership, which is particularly suitable for medical images where tissue boundaries may not be well defined. The module also provides the option to save both the clustered image and the enhanced image, enabling users to store intermediate and final processing results for future reference or external evaluation. This added functionality ensures that the outputs of the clustering process can be efficiently archived or used in subsequent processing steps.

### 2.3. Fuzzy Processing

The Fuzzy Processing module introduces advanced fuzzy image analysis capabilities. This section allows users to generate both the fuzzy edge map and the linked fuzzy edge map, which aim to better handle the uncertainty and fragmented edges commonly encountered in medical imaging. The algorithm uses a simplified fuzzy edge detection approach that combines Gaussian smoothing, gradient magnitude computation, and a fuzzy-inspired non-linear mapping.

The fuzzy edge representation is computed using a Gaussian-based smoothing and a fuzzy membership transformation, while the linked fuzzy edge provides enhanced continuity by applying fuzzy edge-linking strategies. An important feature of this module is the ability to save both the fuzzy edge image and the linked fuzzy edge image for further analysis or documentation. This option enhances the traceability and reproducibility of the results.

### 2.4. Key Features and Workflow

The application currently operates exclusively with square-shaped images. Images that do not meet this criterion are automatically padded with black pixels to achieve the required dimensions.

When the application is started, only the button Load image is enabled and allows the user to import a specific image into the application. A dialog box is provided to assist the user in selecting the desired image file type (e.g., .jpg, .tif, .png). After the image is loaded, all buttons that access the modules of the application are enabled.

When the image is visible (in the first image placeholder), edges will be created and displayed in the next four image holders by clicking the Generate Edges button. The default algorithms for edge extraction are Prewitt, Roberts, Sobel, and Canny for the second placeholders. Along with edge generation, charts of fuzzy metrics such as fuzzy false negatives, fuzzy false positives, fuzzy true positives, and fuzzy index are created and presented (in the lower part of the window) and are computed using the Canny edge as ground truth for all four types of edges presented in the upper part of the window.

The current version extends the *iMIA* application into a larger platform by including fuzzy approaches. MATLAB libraries and tools specifically fuzzy employed in the development of the *iMIA* application are included in Table 1.

**Results presentations.** The main application window is organized by presenting two rows of placeholders. Numerical results are presented in the second row of the application window, displayed as charts with clearly labeled data points to enhance the readability and interpretation of the results.

### 2.5. Pre-Processing Medical Dataset

All the dataset images are in TIF format. To optimize processing time, these files were resized to 512×512 pixels using MATLAB. The *iMIA* application is compatible with multiple image formats, including PNG, JPG, BMP, and TIF. Upon loading, the application retains a copy of the original image, which is subsequently converted to a single-channel (grayscale) format to facilitate edge detection and further processing. For most processing modules within *iMIA*, grayscale images are utilized, with the exception of the Zoom and Edge module, where the original color information is preserved to enhance visualization.

### 2.6. Fuzzy Index

The fuzzy index FI (Equation (1)) is further used for ACO image edge-detection results; the Canny edge is considered as the ground truth based on [53,54].

(1) $$FI=\frac{1}{\left(m\cdot n\cdot max\{\right|N_{i}\left|,\right|N_{d}\left|\}\right)}\cdot \left(|FTP\right|\cdot \sum_{1\leq i\leq m,1\leq j\leq n}\frac{1}{1+FD(i,j)}-\left|FFP\right|-|FFN|).$$

where for two given images $N_{d}=\left\{d_{11},d_{12},d_{13},\ldots ,d_{mn}\right\}$ and $N_{i}=\left\{i_{11},i_{12},i_{13},\ldots ,i_{mn}\right\}$, with *m* and *n* the matrix image dimensions:

- The fuzzy Euclidean distance matrix $FD_{mn}\left(N_{i},N_{d}\right)$ (Equation (2)).
  (2) $$FD\left(N_{i},N_{j}\right)=\frac{D(i,j)}{maxD};$$
- The Euclidean distance between two pixels $N_{i}\left(x_{1},y_{1}\right)$ and $N_{d}\left(x_{2},y_{2}\right)$ (Equation (3));
  (3) $$D\left(N_{1},N_{2}\right)=\sqrt{{\left(x_{2}-x_{1}\right)}^{2}+{\left(y_{2}-y_{1}\right)}^{2}};$$
- Maximum Euclidean distance between two elements in D (Equation (4))
  (4) $$maxD=\sqrt{{\left(m-1\right)}^{2}+{\left(n-1\right)}^{2}};$$
- The scalar cardinality of images sets $N_{i}$ and $N_{d}$ (Equation (5))
  (5) $$|N_{d}|=\sum_{0\leq i\leq m,1\leq j\leq n}N_{d}\left(i,j\right);\left|N_{i}\right|=\sum_{0\leq i\leq m,1\leq j\leq n}N_{i}\left(i,j\right);$$
- The fuzzy true positive FTP, the fuzzy intersection of $N_{i}$ and $N_{d}$, and its scalar cardinality, |FTP| (Equation (6))
  (6) $$FTP=min\left\{N_{i}\left(i,j\right),N_{d}\left(i,j\right)\right\};\left|FTP\right|=\sum_{0\leq i\leq m,1\leq j\leq n}FTP\left(i,j\right);$$
- The fuzzy false positive FFP bounded difference between $N_{i}$ and $N_{d}$ and its scalar cardinality, |FFP| (Equation (7))
  (7) $$FFP=max\left\{0,N_{i}\left(i,j\right)-N_{d}\left(i,j\right)\right\};\left|FFP\right|=\sum_{0\leq i\leq m,1\leq j\leq n}FFP\left(i,j\right);$$
- The fuzzy false negative FFN bounded difference between $N_{d}$ and $N_{i}$ and its scalar cardinality, |FFN| (Equation (8))
  (8) $$FFN=max\left\{0,N_{d}\left(i,j\right)-N_{i}\left(i,j\right)\right\};\left|FFN\right|=\sum_{0\leq i\leq m,1\leq j\leq n}FFN\left(i,j\right);$$

## 3. Results

This section illustrates the use of the *innovative Medical Image Analyzer* (*iMIA*) while testing the considered medical dataset, a selection of magnetic resonance imaging (MRI) cancer images  [62,63] (Section 3.1, including its fuzzy approaches).

### 3.1. Medical Image Dataset Processing

The dataset includes images corresponding to 110 patients initially collected from The Cancer Imaging Archive (TCIA) National Cancer Institute [62,63]. These patients were included in The Cancer Genome Atlas (TCGA) lower-grade glioma collection, for which both fluid-attenuated inversion recovery (FLAIR) sequences and genomic cluster data are available.

**Figure 2:**
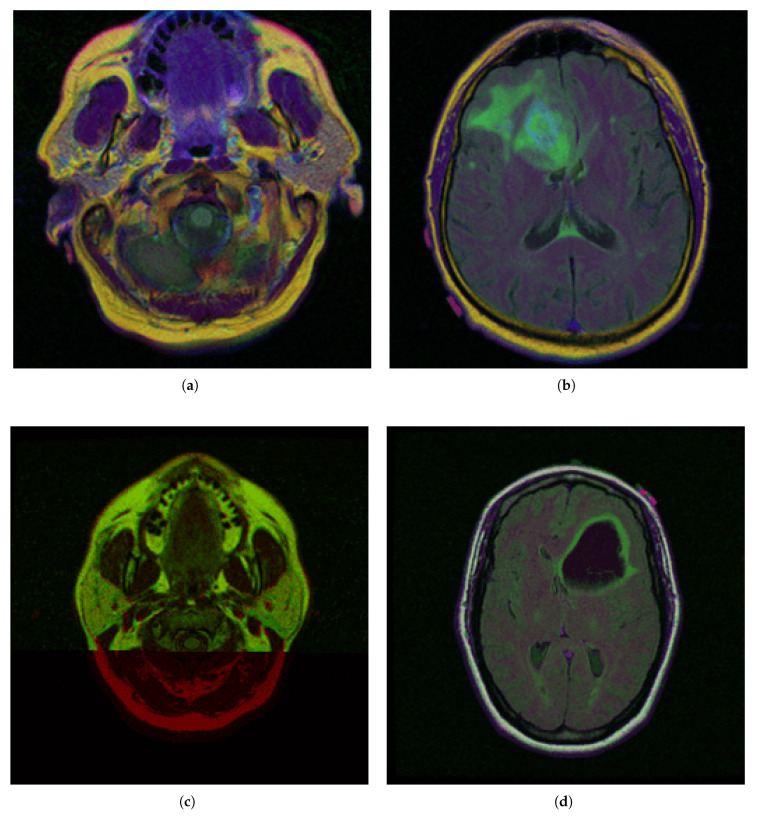
Selected medical MRI dataset from The Cancer Imaging Archive (TCIA) [62] and certified by Duke University experts [63]: (**a**) TCGA_CS_4941_19960909_3 (CS1), (**b**) TCGA_CS_4941_19960909_14 (CS2), (**c**) TCGA_DU_5872_19950223_1 (DU1), (**d**) TCGA_DU_5872_19950223_35 (DU2).

To demonstrate the *iMIA* application’s work with fuzzy approaches, the MRI images TCGA_CS_4941_19960909_3, TCGA_CS_4941_19960909_14 and two images, segmentation masks for FLAIR abnormality based on TCIA [62] and approved by a certified radiologist at Duke University [63], TCGA_DU_5872_19950223_1 and TCGA_DU_5872_19950223_35 (see Figure 2), were chosen The MRI images are further referenced using the abbreviations: CS1, CS2 and DU1, DU2, respectively.

In the segmentation masks (e.g., CGA_CS_4941_19960909_3_mask.tif), different colors represent distinct anatomical or pathological regions:

- Red (255, 0, 0)—Tumor core (non-enhancing tumor + necrotic core)
- Green (0, 255, 0)—Edema (swelling around the tumor)
- Blue (0, 0, 255)—Enhancing tumor (region showing contrast enhancement)

### 3.2. Hardware and Software

In order to extend and test the *i*MIA application, a computer with a 12th Gen Intel(R) Core(TM) i3-1215U 1.20 GHz computer processor (Intel, Santa Clara, CA, USA) was used. The first version of the *innovative Medical Image Analyzer*, *iMIA,* application was presented in [46].

The *iMIA* executable file is available at no cost for testing and evaluation purposes (*innovative Medical Image Analyzer iMIA* executable file, online at https://github.com/cristina-ticala/iMIAv2/ (accessed on 14 July 2025)).

### 3.3. Running Time

The execution time for the Ant Colony Optimization (ACO) algorithm was, on average, 40 s per image, while the Canny edge detection algorithm demonstrated significantly faster performance, with an average processing time of approximately 200 ms per image.

### 3.4. Formal Specification of Parameters

Similar Ant Colony Optimization (ACO) parameters to those reported in studies [50,64] are employed in this work. For instance, when processing images with a resolution of 128×128 pixels, the algorithm is configured as follows:

- To run 1536 iterations for each of the four optimization stages;
- The number of ants is set equal to the Fimage resolution;
- The pheromone matrix is initially set to a low value of 0.0001.

The transition rule is influenced by the balance between pheromone trails and heuristic information, controlled by

- Parameters (1,0.01) which reflect the relative importance of pheromone concentration in guiding the ants’ path selection;
- Locally, pheromone evaporation is applied with a rate of 0.1;
- The global pheromone update incorporates a decay coefficient of 0.001.

The stopping criterion for the algorithm is defined by reaching the maximum number of iterations.

## 4. Discussions

Based on the initial medical MRI dataset (Figure 2), the following section presents the tests performed and the corresponding results obtained throughout the research process. Table 2 presents fuzzy performance indices for all four ACO-based detection methods with diverse operators (ACO_KH, ACO_Chi, ACO_sin, and ACO_pow) applied to multiple case studies. To ensure the reproducibility and comparability of the results, we selected fuzzy-related evaluation metrics such as the fuzzy true positives (FTP), fuzzy false negatives (FFN), fuzzy false positives (FFP), and fuzzy index (FI), as presented and validated in [53,54]. All these collectively provide a comprehensive assessment of edge detection quality relative to the Canny edge, considered the ground truth in this study.

*Statistical analysis* includes descriptive statistics (with mean and standard deviation) for FTP, FFN, FFP, and FI across methods; variance analysis to understand variability and reliability; fuzzy set methods; and alpha-cuts to incorporate the fuzzy nature of the data and related confidence intervals (Table 3).

For all cases, the ACO_pow algorithm achieved the highest fuzzy true positive rates and the lowest rates of fuzzy false negatives and fuzzy false positives. These facts are reflected in the significantly higher fuzzy index (FI). Although ACO_pow produces edges with fewer pixels, making them numerically closer to the Canny edge used as ground truth, a visual assessment of the results indicates that the edges generated by ACO_sin appear more continuous and perceptually complete.

Moreover, based on the results presented in Table 2, after ACO_pow, the next most effective algorithm is consistently ACO_sin. Across all case studies, ACO_sin demonstrates stable and competitive performance, typically achieving the second-highest fuzzy true positive (FTP) values and fuzzy index (FI) scores among the evaluated methods. This suggests that, despite the lower performance in fuzzy indexes, ACO_sin is capable of providing a more coherent and visually accurate edge representation, enhancing its practical relevance in medical image analysis.

Therefore, ACO_Sin can be considered a reliable alternative, particularly when a balance between computational efficiency and edge detection accuracy is desired. It offers a solid compromise, achieving better performance than the other classic ACO variants while remaining slightly less computationally intensive compared to ACO_pow.

**Figure 3:**
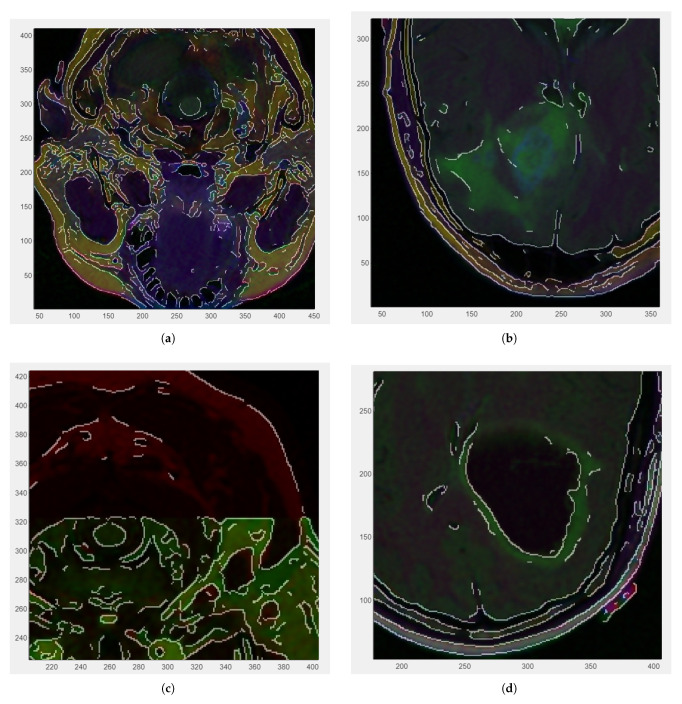
Zoomed-in RGB images with Canny edge white overlaid: (**a**) CS1 (zoom factor 1.3) 0.3 cm. (**b**) CS2 (zoom factor 1.4). (**c**) DU1 (zoom factor 1.7) 0.2 cm. (**d**) DU2 (zoom factor 1.4).

Figure 3 illustrates zoomed-in sections of the four medical images included in the dataset, each overlaid with the Canny edge for reference. The visualizations offer a detailed perspective on the edge distribution within localized regions of the images, providing a closer examination of anatomical boundaries. The Canny edge is displayed as a fine contour that assists in assessing the accuracy and relevance of the detected edges.

Additionally, the interface includes an adjustable zoom slider and an overlay toggle, which facilitate dynamic interaction with the processed images, enabling users to inspect fine details and edge alignment with precision. These visual tools support a comprehensive and intuitive analysis of edge detection performance across different regions of interest.

**Table 2:** Results of the fuzzy indexes with Canny edge image as ground truth and ACO edges on four different operators: KH, Chi, Sin, and Pow [49,50] (FFN—fuzzy false negatives; FFP—fuzzy false positives; FTP—fuzzy true positives, FI—fuzzy index; see Section 2.6). Results are represented in iMIA application’s charts as seen in Figure 4.

Case Study	%FTP	%FFN	%FFP	%FI
TCGA_CS_4941_19960909_3				
ACO_KH	13,813.5558	78.9893	2545.7823	0.8410
ACO_Chi	13,830.1532	78.5887	2529.1849	0.8420
ACO_Sin	13,808.6418	78.8579	2550.6963	0.8407
ACO_Pow	15,490.0836	53.9731	869.25447	0.9440
TCGA_CS_4941_19960909_14				
ACO_KH	14,584.0047	35.722	1786.1172	0.8886
ACO_Chi	14,585.6887	36.0614	1784.4331	0.8887
ACO_Sin	14,587.8412	35.9304	1782.2807	0.8888
ACO_Pow	15,641.7746	33.7812	728.34729	0.9537
TCGA_DU_5872_19950223_1				
ACO_KH	14,646.0902	39.7504	1724.4969	0.8925
ACO_Chi	14,658.9086	40.0822	1711.6785	0.8932
ACO_Sin	14,656.1966	39.7628	1714.3904	0.8931
ACO_Pow	15,794.2675	33.4020	576.31957	0.9631
TCGA_DU_5872_19950223_35				
ACO_KH	15,211.4221	23.1851	1163.4043	0.9275
ACO_Chi	15,212.5014	23.1170	1162.3249	0.9276
ACO_Sin	15,212.4069	23.2139	1162.4195	0.9276
ACO_Pow	15,826.9928	22.4937	547.83357	0.9653

**Table 3:** Descriptive statistics: mean; standard deviation (std); variance analysis; fuzzy set methods (alpha-cuts); and confidence intervals to quantify uncertainty.

Statistics	%FTP	%FFN	%FFP	%FI
Mean	14,236	72.60	2123.70	0.8669
Std	836.37	12.42	836.37	0.0514
Variance	699,510	154.27	699,510	0.0026
Alpha-cut intervals				
Alpha = 0.00	[13,399.24, 15,071.98]	[60.18, 85.02]	[1287.36, 2960.10]	[0.82, 0.92]
Alpha = 0.25	[13,511.29, 14,959.92]	[61.85, 83.36]	[1399.41, 2848.04]	[0.82, 0.91]
Alpha = 0.50	[13,644.21, 14,827.01]	[63.82, 81.38]	[1532.33, 2715.13]	[0.83, 0.90]
Alpha = 0.75	[13,817.42, 14,653.79]	[66.39, 78.81]	[1705.55, 2541.91]	[0.84, 0.89]
Alpha = 1.00	[14,235.61, 14,235.61]	[72.60, 72.60]	[2123.73, 2123.73]	[0.87, 0.87]
95% Confidence Interval	[12,904.76, 15,566.46]	[52.84, 92.37]	[792.88, 3454.58]	[0.79, 0.95]

**Figure 4:**
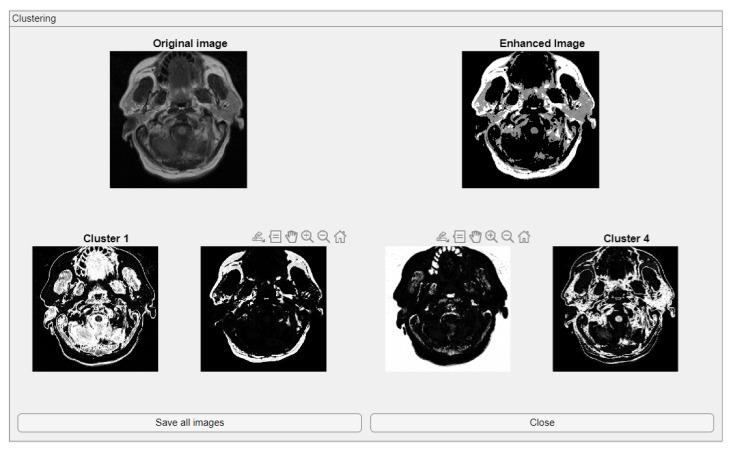
Clustering with fuzzy C-means (FCM) results within iMIA platform: The clusters emphasize various structural and textural components, with some clusters isolating specific regions with clear edges and others focusing on more homogeneous areas. The segmentation reveals critical areas with varying intensity patterns, possibly indicative of different tissue types or pathological regions: TCGA_CS_4941_19960909_3 (CS1) (additional results are shown in Figure A1).

The comparative charts and visual presentation of edges allow for a detailed analysis of each algorithm’s strengths and weaknesses, offering insight into the trade-offs between edge completeness and false detection rates. Figure 4 illustrates the fuzzy C-means (FCM) clustering results obtained from the four case studies: CS1, CS2, DU1, and DU2. Each subfigure (A, B, C, and D) presents the following sequence:

- **Original Image**: The initial input image from the dataset.
- **Enhanced Image**: The result after applying image enhancement techniques to improve contrast and feature visibility, likely to support better clustering outcomes.
- **Clusters 1 to 4**: The segmentation outputs, where the image has been partitioned into four distinct clusters. Each cluster highlights different regions based on pixel similarity, potentially corresponding to anatomical structures, tissues, or specific features of interest within the medical images.

**Figure A1:**
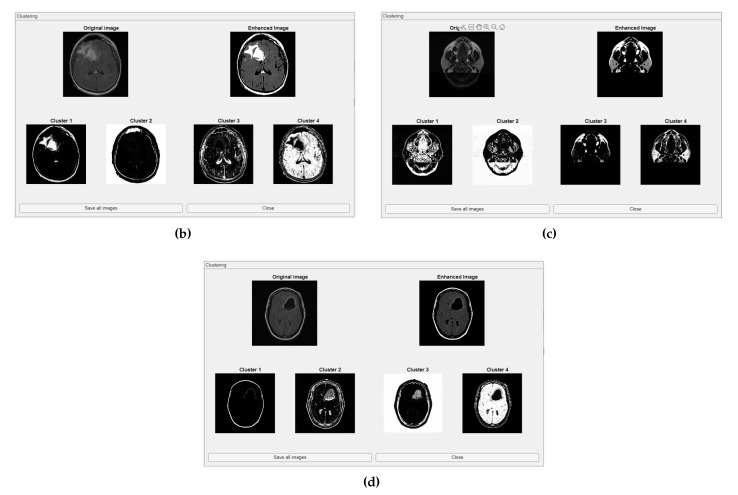
Clustering with fuzzy C-means (FCM) results within *iMIA* platform. (**b**) TCGA_CS_4941_19960909_14 (CS2), (**c**) TCGA_DU_5872_19950223_1 (DU1), (**d**) TCGA_DU_5872_ 19950223_35 (DU2) (refer to the image (**a**) CS1 result in Figure 4).

**Key Observation**: Figure 4 and Figure A1 collectively showcase the clustering module’s capacity to aid in visual discrimination and structural interpretation across the studied medical cases.

**Figure 5:**
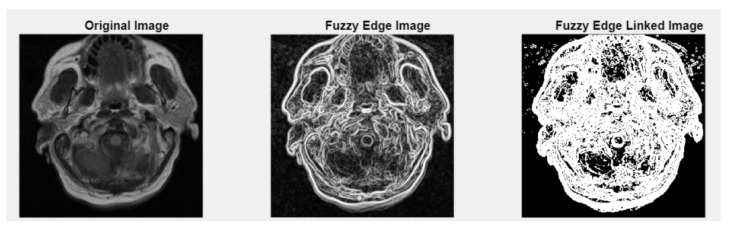
Fuzzy edge results from *iMIA* (Gaussian smoothing, gradient magnitude computation, a fuzzy-inspired non-linear mapping): TCGA_CS_4941_19960909_3 (CS1). Leftmost image: original grayscale image; middle image: fuzzy edge; rightmost image: linked fuzzy edge (additional results are shown in Figure A2).

**Figure A2:**
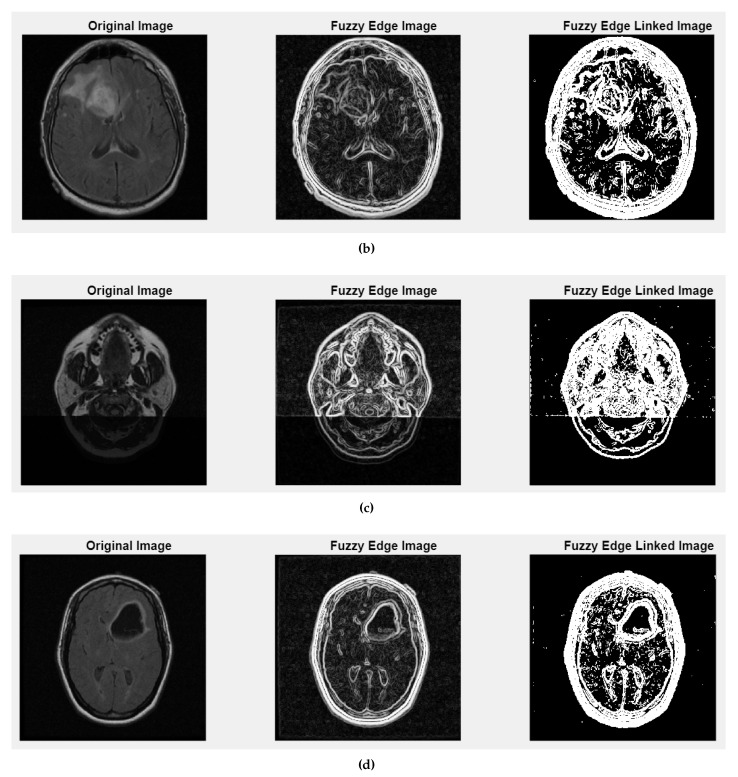
Fuzzy edge results from *iMIA* (Gaussian smoothing, gradient magnitude computation, a fuzzy-inspired non-linear mapping): (**b**) TCGA_CS_4941_19960909_14 (CS2), (**c**) TCGA_DU_5872_19950223_1 (DU1), (**d**) TCGA_DU_5872_19950223_35 (DU2). Leftmost image: original grayscale image; middle image: fuzzy edge; rightmost image: linked fuzzy edge (refer to the image (**a**) CS1 result in Figure 5).

Figure 5 and Figure A2 illustrate the fuzzy edge detection approach in which Gaussian smoothing, gradient magnitude computation, and a fuzzy-inspired non-linear mapping are combined for the four case studies CS1, CS2, DU1, DU2. In each subfigure, the leftmost panels display the original (grayscale) scans, where the tissue boundaries naturally exhibit a degree of fuzziness due to partial volume effects, image noise, and inherent anatomical ambiguity. The middle panels present the application of fuzzy edge detection algorithms, which are specifically designed to preserve the uncertainty associated with boundary localization.

Unlike conventional edge detection methods that impose hard, binary edge definitions, these fuzzy approaches allow for gradual transitions and probabilistic boundary representations, which are particularly relevant for medical imaging contexts. The rightmost panels showcase the results obtained after applying fuzzy linked edge techniques. These methods focus on establishing connections between detected edge segments by considering similarity measures, spatial proximity, and continuity constraints. As a result, fragmented or weak edges are probabilistically linked, producing more continuous and visually coherent boundary structures while still preserving the underlying uncertainty.

This progression from the original image to the fuzzy edge detection, and finally to the fuzzy-linked edge representation, reflects a complete processing workflow that supports both edge preservation and structural completeness. Such an approach is highly beneficial when analyzing complex medical images where exact boundary definitions are not always feasible and maintaining a degree of flexibility in edge interpretation is essential.

The main clinical implications in high-stakes contexts of fuzzy logic in medical decision making include enhanced handling of uncertainty, improved diagnostic support, flexible and intuitive modeling, and integration of expert knowledge. Limitations and major challenges in high-stakes contexts of fuzzy logic in medical decision making include lack of standardization, validation and regulatory approval, complexity and transparency, potential misinterpretation, limited evidence database, and nevertheless ethical and legal concerns.

### Additional Tests: U-Net and Numerical Comparison with ACO

We implemented a custom U-Net [55] architecture tailored for edge detection with an encoder–decoder structure enhanced by skip connections. The encoder uses progressively deeper convolutional layers, and the decoder restores spatial resolution through transposed convolutions. Weighted pixel classification was applied to address class imbalance.

**Figure 6:**
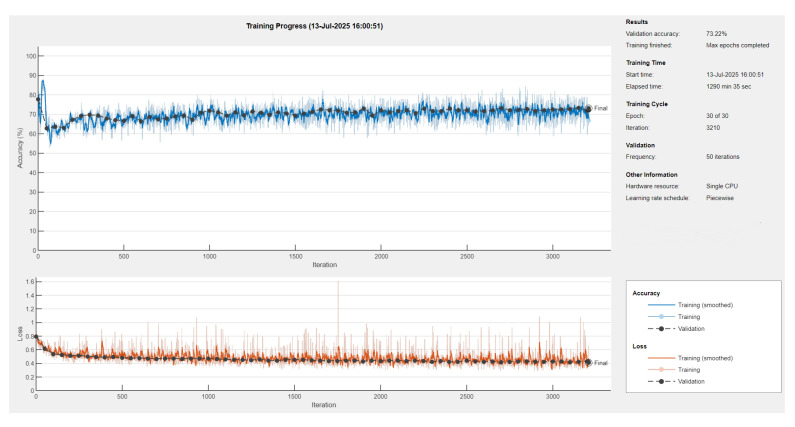
Training progress of U-Net neural network with ResNet18 encoder-decoder [65].

The model was trained (see Figure 6) with appropriate augmentation and class balancing to boost edge detection accuracy.

**Figure 7:**
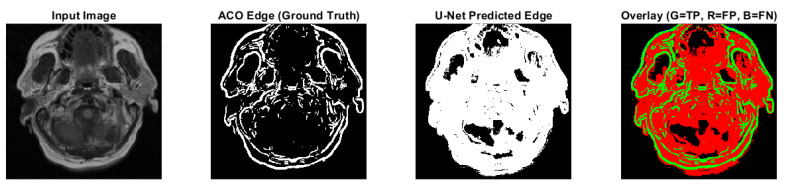
CS1 results when comparing ACO and U-Net (Red: U-Net Predicted Edge, Green: ACO Edge; additional results are shown in Figure A3).

The U-Net model was trained on a dataset of 1074 images using the Adam optimizer with an initial learning rate of $3\times 10^{-4}$. A piecewise learning rate schedule was applied, reducing the learning rate by half every 10 epochs to stabilize learning over time. Training was conducted for up to 30 epochs with a mini-batch size of eight, and the model was evaluated on a validation set every 50 iterations (Figure 7). The training was shuffled every epoch and set to run on the available computing environment: a CPU. Early stopping was enabled with a validation patience of 10, allowing training to halt if no improvement was observed.

Further, a pretrained ResNet-18 encoder–decoder [65] was integrated to improve feature extraction and accelerate convergence (Table 4).

Predicted edges from the U-Net with ResNet18 encoder–decoder differ in thickness compared to the ACO-derived ground truth across test cases. In some cases, the predicted edges are significantly thicker (over-segmented), while in others, they are fewer or thinner (under-segmented). This variability affects the similarity metrics as follows:

- **High recall values** (e.g., 0.9880 and 0.9963 for CS1 and DU1 images) indicate that in these cases, the model successfully identified most ground truth edge pixels—though often with excessive prediction (over-segmentation);
- **Very low recall** (e.g., 0.0483 for DU2 image) shows that in some cases, the model fails to detect most of the true edge pixels—under-segmentation;
- **Low precision values** across all cases (from 0.0952 to 0.2342) suggest that many of the predicted edge pixels do not match the ground truth, indicating high false positive rates;
- **F1-score and Jaccard Index** are low overall (F1 from 0.0641 to 0.3786; Jaccard from 0.0331 to 0.2335), pointing to a general mismatch between predicted and true edge locations;
- **SSIM and Max NCC** vary from moderate to low (SSIM up to 0.7892, NCC mostly below 0.33), reflecting inconsistent structural similarity and limited pixel-level correlation across cases.

The U-Net model can successfully detect edge regions but shows inconsistent behavior across cases—over-predicting in some and under-predicting in others. This results in imprecise edge localization. Future improvements will focus on training refinement, edge-aware loss functions, and post-processing steps such as edge thinning to enhance precision and structural accuracy.

## 5. Conclusions

Medical image analysis platforms are typically designed to support both fundamental and advanced processing tasks. The proposed iMIA platform effectively addresses a broad spectrum of these tasks, providing fast and reliable results. For future extensions, integrating deep learning (DL) models pre-trained on large and diverse datasets holds significant potential to further enhance the accuracy and robustness of the outcomes.

Based on the computed fuzzy index values, the Ant Colony Optimization (ACO) technique demonstrates competitive performance, yielding results that are comparable to those achieved by the Canny edge detection algorithm across the analyzed cases. The edges obtained through the ACO approach exhibit a distinctive balance between completeness and softness. While they may not always produce the crisply defined boundaries characteristic of traditional methods like Canny, ACO edges tend to offer visually coherent structures. This makes them particularly valuable in medical imaging contexts where anatomical boundaries are inherently fuzzy or where noise and low contrast can lead classical methods to miss or fragment subtle edges. The ACO edges, therefore, present a competitive alternative by preserving more of the natural variability and uncertainty present in real tissues, offering a more clinically realistic representation.

The iMIA platform places a strong emphasis on fuzzy logic-based processing, which provides a valuable framework for managing the inherent uncertainty and imprecision present in medical imaging. Unlike traditional hard-boundary methods, fuzzy edge detection and fuzzy-linked edge techniques used within the platform preserve soft transitions and ambiguous boundaries characteristic of medical scans, particularly in areas affected by noise, partial volume effects, and low contrast.

The incorporation of fuzzy processing enables the platform to deliver more nuanced and clinically meaningful edge representations, offering smoother, more connected edge structures while maintaining the ability to express uncertainty. This is especially beneficial in complex anatomical regions where strict binary decisions may fail to capture subtle tissue variations. The clustering module further enhances the analytical capability of the platform. The clustering process successfully segments the images into meaningful subregions, revealing distinctive patterns and boundaries that are not easily discernible in the original images. The enhanced images produced during this process offer sharper contrast, facilitating clearer cluster separation and supporting more accurate subsequent analysis. This step adds significant value to the overall image analysis workflow by highlighting areas of interest that might otherwise remain hidden.

Looking forward, the integration of fuzzy logic with machine learning models represents a promising direction for future development. Combining the adaptability and interpretability of fuzzy methods with the learning capacity of data-driven approaches could result in more powerful and reliable tools for medical image analysis. Further development will focus on increasing the flexibility of the platform by enabling the selection and overlay of various edge types on the original image across all application modules. Additionally, upcoming improvements will include adding fuzzy edges as selectable options within the dropdown menus and developing a dedicated ACO edge linking algorithm, analogous to the fuzzy edge linking approach, to further enhance the quality and continuity of the extracted edges.

Overall, the following synthesis of conclusions highlights the research results related to the achievement of the proposed objectives.

- Ant Colony Optimization is a strong edge detection method. It performs on par with the Canny edge detector, as expected;
- ACO achieves edges without relying on additional edge linking algorithms, detecting even subtle discontinuities;
- The edges are following the true content of the image, avoiding artificial or forced connections;
- While not as sharp as Canny edges, ACO edges maintain continuity and represent fuzzy, uncertain anatomical boundaries;
- Fuzzy logic is central to the iMIA processing approach; fuzzy edge detection handles uncertainty and imprecision well in medical imaging, and it preserves soft transitions and ambiguous regions;
- The clustering module segments images into meaningful sub-regions, enhances contrast, and reveals patterns not obvious in the original images.
- A customized U-Net model is being developed for edge extraction, adapting it from its original segmentation purpose; although in the early stages, it shows promise. Here, ACO currently remains a more reliable edge detection method.

### Future Improvements

Deep learning and especially convolutional neural networks (CNNs) and nnUNET [66,67,68], when combined with fuzzy C-means and ACO, could concretely improve the following:

- Clustering accuracy while automatically learning hierarchical and discriminative images features;
- Image segmentation in large or noisy image datasets;
- Workflow speed while automatically segment and classify medical images;
- Interpretability and trustworthy outputs as DL could incorporate uncertainty estimation to help doctors feel more confident in their diagnoses.
- Edge detection techniques can be applied to MRI or CT brain images, including for highlighting fluid diffusion associated with Alzheimer’s disease. Specific Alzheimer’s disease-related use cases:
  – Ventricular enlargement: edge detection may help outline and measure these ventricles without full segmentation;
  – Cortical atrophy: edges between cerebrospinal fluid and cortical gray matter become more pronounced;
  – Midline shift ar asymmetry detection: Early signs of structural imbalance can be highlighted through edge-based symmetry analysis.
- The types of edge detection which can be used are the following:
  – Sobel, Prewitt, Roberts, Canny, ACO variants;
  – The edge detection methods can be used in combination with filtering techniques to reduce noise.

Edge detection in Alzheimer’s imaging

- Is not a substitute for segmentation but serves as a valuable, lightweight tool for early structural analysis;
- Enhances anatomical visualization and helps identify atrophic patterns;
- Can be integrated into preprocessing pipelines for diagnostic support, data annotation, or machine learning model input;
- Must include images of good quality that are properly preprocessed;
- Indicates that edge detection is often just one step in the analysis process, and specialized interpretation is needed to differentiate between pathological fluid diffusion and normal structures or imaging artifacts.

## Figures and Tables

**Table 1:** Specific fuzzy utilities used with *iMIA*.

Fuzzy Tools	Utility
Fast fuzzy C-means image segmentation	Segment N-dimensional grayscale images into classes using efficient C-means or fuzzy C-means clustering algorithm.
Fuzzy Logic Toolbox	Fuzzy Logic Toolbox provides MATLAB functions, apps, and a Simulink block for analyzing, designing, and simulating fuzzy logic systems.

**Table 4:** Performance metrics comparing ACO ground truth and U-Net (ResNet18) predicted edges.

Metric	CS1	CS2	DU1	DU2
ACO Edge Pixels (%)	14.12%	9.75%	9.19%	5.52%
U-Net Predicted Edge Pixels (%)	59.56%	15.58%	47.56%	2.80%
Accuracy	0.5422	0.8065	0.6157	0.9221
Precision	0.2342	0.1920	0.1926	0.0952
Recall	0.9880	0.3067	0.9963	0.0483
F1-Score	0.3786	0.2361	0.3229	0.0641
Jaccard Index	0.2335	0.1339	0.1925	0.0331
Dice Coefficient	0.3786	0.2361	0.3229	0.0641
SSIM	0.3292	0.5918	0.4883	0.7892
Max NCC	0.3281	0.2217	0.3322	0.1965

**Figure A3:**
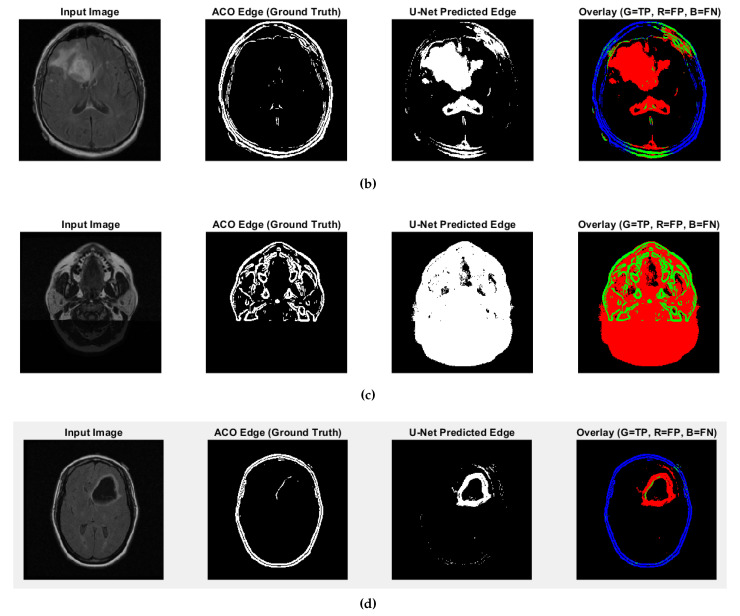
Fuzzy edge results from *iMIA* (results of comparing ACO and U-Net): (**b**) TCGA_CS_4941_ 19960909_14 (CS2), (**c**) TCGA_DU_5872_19950223_1 (DU1), (**d**) TCGA_DU_5872_19950223_35 (DU2) (Red: U-Net Predicted Edge, Green: ACO Edge; refer to the image (**a**) CS1 result in Figure 7).

## Data Availability

The installer of iMIA presented in the study is openly available at https://github.com/cristina-ticala/iMIAv2/ accessed on 14 July 2025.

## Abbreviations

   The following abbreviations are used in this manuscript:

TCIA	The Cancer Imaging Archive
iMIA	innovative Medical Image Analyzer
FCM	Fuzzy C-Means
DL	Deep Learning
ACO	Ant Colony Optimization
CNN	Convolutional Neural Networks
FTP	Fuzzy True Positives
FFN	Fuzzy False Negatives
FFP	Fuzzy False Positives
FI	Fuzzy Index
